# Optimizing evidence-based practice implementation: a case study on simulated patient protocols in long-term opioid therapy

**DOI:** 10.1186/s43058-024-00575-y

**Published:** 2024-04-22

**Authors:** Ellen Green, Megan Hamm, Catherine Gowl, Reed Van Deusen, Jane M. Liebschutz, J. Deanna Wilson, Jessica Merlin

**Affiliations:** 1 College of Health Solutions, Arizona State University, Tempe, AZ USA; 2 School of Medicine, University of Pittsburgh, Pittsburgh, PA USA; 3 Perelman School of Medicine, University of Pennsylvania, Philadelphia, USA

## Abstract

**Background:**

Substantial work has been done to update or create evidence-based practices (EBPs) in the changing health care landscape. However, the success of these EBPs is limited by low levels of clinician implementation.

**Objective:**

The goal of this study is to describe the use of standardized/simulated patient/person (SP) methodology as a framework to develop implementation bundles to increase the effectiveness, sustainability, and reproducibility of EBPs across health care clinicians.

**Design:**

We observed 12 clinicians’ first-time experiences with six unique decision-making algorithms, developed previously using rigorous Delphi methods, for use with patients exhibiting concerning behaviors associated with long-term opioid therapy (LTOT) for chronic pain. Clinicians were paired with two SPs trained to portray individuals with one of the concerning behaviors addressed by the algorithms in a telehealth environment. The SP evaluations were followed by individual interviews, guided by the Consolidated Framework for Implementation Research (CFIR), with each of the clinician participants.

**Participants:**

Twelve primary care clinicians and 24 SPs in Western Pennsylvania.

**Main measurement:**

The primary outcome was identifying likely facilitators for the successful implementation of the EBP using the SP methodology. Our secondary outcome was to assess the feasibility of using SPs to illuminate likely implementation barriers and facilitators.

**Results:**

The SP portrayal illuminated factors that were pertinent to address in the implementation bundle. SPs were realistic in their portrayal of patients with concerning behaviors associated with LTOT for chronic pain, but clinicians also noted that their patients in practice may have been more aggressive about their treatment plan.

**Conclusions:**

SP simulation provides unique opportunities for obtaining crucial feedback to identify best practices in the adoption of new EBPs for high-risk patients.

**Setting:**

Zoom simulated patient evaluations.

Contributions to the literature

- This study uses simulated patients (SPs) in implementation science planning, offering insights into identifying gaps and tailoring implementation strategies effectively.
- Focusing on long-term opioid therapy, our research exemplifies SPs’ practical role in implementing evidence-based practices, addressing a critical gap in substance use therapy.
- Beyond training, our findings provide insights into SPs as facilitators for professionals dealing with high-risk patients, acknowledging both the potential benefits and limitations of the SP methodology.

## Introduction

Timely adoption of current evidence-based practices (EBPs) is key to ensuring high-quality care in our changing health care environment. Creating EBPs alone is insufficient to ensure their implementation. Without well-designed implementation strategies, the adoption of these practices can take decades [1]. This is because clinicians often face barriers to implementing EBPs, including limited awareness, resistance to change, and resource constraints. Organizational culture, patient factors, and the complexity of implementation further contribute to the challenges. Evaluation of implementation strategies outside of an active practice setting can address these barriers and increase the likelihood of dissemination, long-term adoption, and appropriate use of EBPs by providing a controlled environment for assessment, feedback, and identification of facilitators for a successful implementation [2–4]. We argue that the standardized/simulated patient/person (SP) methodology serves as a valuable tool for formulating implementation strategies for EBPs before their application in practice.

SPs are people trained to portray complex behaviors and react as an actual patient would to a clinician in real time creating a fully interactive patient-clinician experience outside of a real-world practice [5]. SPs can be trained to consistently exhibit specific emotions (e.g., anger [6]), desires (e.g., prescriptions), and/or patient needs (e.g., language barriers [7]) across clinicians. The flexible nature of simulation can be leveraged to reflect either a single patient encounter or multiple patient visits portraying the passage of time depending on the application (e.g., teach providers how to perform a physical exam or re-evaluate patients after a new prescription). While SP methodology is commonly used to train and test clinicians on new techniques [5, 8–15], its application to the planning phases of implementation science remains limited. Our work specifically leverages SP methodology within the planning phases of an implementation bundle for an EBP - a novel approach that has been underutilized in existing literature.

There are several advantages to using SP methodology as a part of implementation strategy. First, the consistent portrayal of a patient case can help identify gaps in EBP implementation and facilitate targeted solutions for future implementation. Second, recruiting clinicians from multiple and diverse practices to use the EBPs with SPs can provide insight into how the EBP would be best implemented in their unique practice setting after the provider has first-hand experience with the EBP. This can provide richer and more diverse insight for implementation scientists relative to feedback from directly implementing an EBP into a singular practice that may not generalize to other clinics.

Likewise, evaluating an EBP outside of the daily activities of a typical clinical practice provides clinicians with immediate and protected time for debriefing. Without dedicated time for good feedback, it is difficult to identify areas of improvement for implementation. Also, developing implementation strategies for EBPs in practice can be high risk for patients. The use of SPs provides a safe environment to develop implementation strategies and gain active experience with EBPs without putting patients at risk [16]. Lastly, SPs can provide insight into events that may be uncommon or take a long time to occur in practice, which can expedite necessary adaptation of implementation strategies for EBPs. Overall, SPs may provide a critical step in increasing the likelihood of a successful adoption of an EBP by identifying the barriers and facilitators prior to implementation in the field.

For these reasons, we adopted the SP methodology for a research project implementing an evidence-based approach to addressing concerning behaviors in patients on long-term opioid therapy (LTOT), such as diversion, use of other substances, or non-adherence to pain therapy. Although the evidence for the effectiveness of LTOT is limited [17–19], there are millions of Americans prescribed opioid analgesics yearly, with more than 17% of Americans receiving an opioid prescription in 2017, with an average of 3.4 opioid prescriptions dispensed per patient [20]. Multiple efforts to improve opioid prescribing have occurred on the broader policy level (e.g., prescription drug monitoring programs), the insurance level (limits on doses or length of time), and through education (the RDA risk evaluation and mitigation strategy program [21] and most recently, the drug enforcement agency requirement for training on addiction and opioids) [22]. While opioid prescribing has decreased overall [23], none of these broader measures address concerning behaviors among patients taking LTOT. To augment non-specific recommendations in the CDC guide to prescribing opioids (“weigh the risks and benefits” [24]) and other broader prescribing policy, our team previously developed a set of evidence-based clinical decision-making algorithms using Delphi process to address concerning behaviors among patients prescribed opioids. The lack of uptake of most clinical guidelines [25, 26] led the team to look for effective ways to implement these EBP. Because the concerning behaviors of patients on LTOT may occur sporadically among primary care physicians (PCPs), using the SP methodology would allow for rapid feedback, making it attractive for developing and testing potential implementation methods of the EBP.

In this article, we describe the SP methodology for developing an implementation bundle for a new EBP to address concerning behaviors among patients on LTOT. In conjunction with the SP methodology, we used observation and discussion from one-on-one structured interviews to develop an implementation bundle to increase the likelihood of effective, sustainable, and reproducible adoption in practice. Our approach was guided by the Consolidated Framework for Implementation Research (CFIR), a commonly used tool to guide qualitative inquiry about how clinicians would implement EBPs in practice [27].

## Methods

**Fig. 1:**
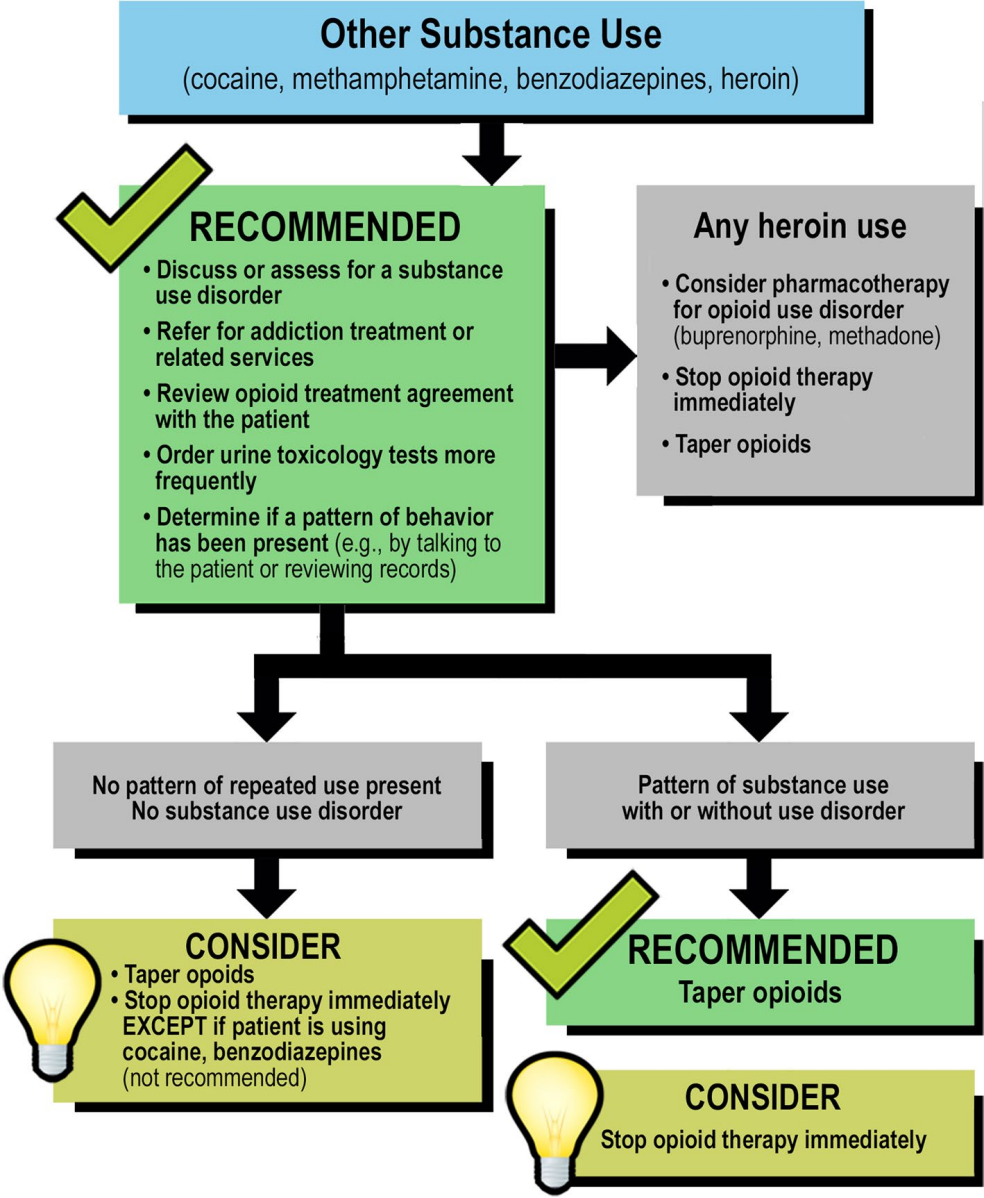
SEQ figure \* ARABIC 1: “Other Substance Use” Algorithm

We demonstrate the important and practical use of the SP methodology for developing implementation strategies for a new EBP: 6 treatment algorithms designed to address common and challenging behaviors associated with long-term opioid therapy (LTOT) developed by Merlin and colleagues and published in 2016 [28]. As previously described, these algorithms were developed using a modified Delphi process [29, 30], a rigorous methodology that uses several rounds of questionnaires sent to a panel of experts to find consensus on how to respond to behaviors such as missing appointments with clinicians prescribing the opioid, taking more opioid than prescribed, and substance use. One of the algorithms is included as an example of the new EBP in Fig. 1. In the present study, we conducted SP sessions with providers using 6 SP cases, one for each algorithm. These SP sessions were followed by one-on-one structured interviews with questions mapping onto domains from the CFIR to assist in the development of an implementation bundle for the new EBP.

### Case development

We developed 6 SP cases. Each case simulated a patient exhibiting a unique concerning behavior addressed by the algorithms (see Table 1 outlining the behaviors portrayed). The SP cases were written with unfolding steps to represent three visits with a provider, because the algorithms guide decision points that would normally occur in subsequent follow-up visits in real-life practice (Fig. 1). The unfolding nature of the scenarios was piloted early in the SP case development process to ensure feasibility.

**Table 1:** SP cases

Algorithm	Case description
**Missing appointments**	*Patient frequently misses appointments. Recent refill request led to the scheduling of a telemedicine appointment today after missing an earlier in-person visit due to transportation issues. Patient is now out of medication and expresses urgency for a refill. History of intermittent attendance despite past reminders.*
**Taking opioids for symptoms other than pain**	*Patient is using oxycodone for symptoms beyond pain management. Seeking a refill during a visit to establish with a new primary care physician. High-stress job overseeing multiple restaurant chains, managing a team, and dealing with calendar issues. Balancing responsibilities as a parent of four boys adds to the stress. Unrecognized anxiety, difficulty falling asleep, and reliance on oxycodone for sleep aid over the past 2 weeks (approximately 10 nights).*
**Using more opioid medication than prescribed**	*Patient requests an early refill of pain meds, indicating potential opioid use disorder by the third visit. More active at home, engaging in renovations, leading to increased oxycodone usage (2–4 times daily instead of the prescribed twice). Patient experiencing heightened stress due to job loss a month ago. Pain typically at 2–3/10, managed without medication, but escalates to 10/10, prompting oxycodone use. Lack of awareness of opioid agreement details and increased frequency of use hint at emerging opioid use disorder.*
**Asking for an increase in opioid dose**	*Patient seeks an increase in opioid dose due to decreasing effectiveness over time. History of gradual dose adjustments with Dr. Williams, starting with short-acting oral morphine and transitioning to long-acting MS Contin. Current dose maintained for 8 months, with previous increases providing relief for 10–12 months each time. Patient familiar with the concept of tolerance and expresses nervousness about a new doctor’s understanding. Patient’s approach the visit is hopeful but tempered by some trepidation.*
**Alcohol use**	*Patient combines alcohol with prescribed opioid medication. Seeking a refill during the first visit to establish with a new primary care physician. Almost out of pills, with about 5 remaining. Pain meds are effective as prescribed, but a positive score on an alcohol use screening questionnaire raises concerns. Patient acknowledges alcohol use as the only "red flag" during the visit.*
**Other substance use**	*Patient uses heroin in addition to prescribed opioid medication. Seeking a refill during the first visit to establish with a new primary care physician. Out of prescribed pills, with the last one taken this morning. Expresses concern that the medication’s effectiveness has decreased and inquires about a higher dose or additional pills. Patient avoids disclosing heroin use and mentions financial challenges in obtaining the medication. Patient may be hesitant about a urine drug screen test but plans to delay it to ensure a clear result. Mild withdrawal symptoms occur if without medication for too long.*

The algorithms created by Merlin, Young, Azari, et al. (2016) also addressed “Aggressive Behavior”; however, based on feedback from the patient-provider advisory board, we did not review the algorithm in the present study due to potential subjective and biased nature of this term with regard to both gender and race

SP cases were next reviewed by a Patient-Provider Advisory Board (PPAB) consisting of 3 patients with lived experience with opioids, 4 researchers (among whom are PCPs familiar with caring for patients with opioid misuse disorder), and a primary care provider with familiarity with providing care for patients with opioid misuse. SP cases were edited based on feedback from the PPAB. In concert with the review of the 6 cases, the PPAB reviewed the instructions which provided context, expectations for SP-clinician interactions, and training on the algorithms (see Appendix). Finally, cases and instructions were piloted with an SP and a provider outside of the panel. During this pilot, a physician with topical expertise was recruited to interact with SPs portraying two SP cases over three subsequent visits on a remote/telehealth platform (Zoom). This pilot helped to further develop the other five SP cases in structuring how clinicians would be oriented, updated, and guided through the simulations.

### Training and description of organization for SPs

Four experienced SPs were recruited from the University of Pittsburgh SP program to portray the patients exhibiting misuse behaviors. The SPs in the University of Pittsburgh School of Medicine SP Program received foundational training in case portrayal, providing feedback, supported physical exam training, and checklist scoring. This 16-h onboarding combines both active training and also guided observation of SP activities. It prepares SPs to identify, recognize, and reward learner skill in portrayal, and to record it faithfully in assessments.

To allow rotation, redundancy and information sharing, the SPs worked in pairs for each case, alternating the role of moderator and patient. When not portraying the patient, the SP acted as a moderator by providing clinicians with inter-visit updates in accordance with what the clinicians ordered in the first session and noted the passage of time between visits. A fifth experienced SP was recruited to proctor the event—orienting the clinicians as they arrived, running the Zoom sessions, and serving as a backup should one of the other SPs not be able to participate. They also were given an overview of case content, portrayal, and event structure. SPs were provided with case materials a week in advance of the portrayal date, were able to ask questions over email, and completed a case-specific training to align portrayal with parameters provided in the inter-visit updates with SP staff in the 45 min preceding the simulation. The SP program follows the Association for Standardized Patient Educators (ASPE) Standards of Best Practice, which “were written to ensure the growth, integrity, and safe application of SP-based education practices.” [9]

### Description of session for clinicians

Clinicians were emailed information and instructions about the event prior to participating in the session (see Appendix). All sessions were held virtually via the Zoom interface due to the COVID pandemic. During the sessions, there was a brief orientation for participants. The orientation included (1) a brief training in how to use the algorithms; (2) an overview of how to approach the simulated interaction (i.e., as close to real practice as possible); and (3) an overview of the one-on-one interview that would follow to discuss the approaches to implement the management algorithms.

Clinicians then moved into Zoom breakout rooms to begin their patient encounters. Clinicians were given up to 60 min to have their 3 distinct visits per patient. There was a 15-min break, and then another 60 min for the second patient scenario.

For each of the 60-min SP scenarios, clinicians were told that they were about to see a patient who was being seen by one of their partners (Dr. Williams) who recently left the practice. Dr. Williams had started the patient on opioid therapy and had an opioid agreement with the patient. Participants were given a copy of Dr. Williams’ last progress note and the opioid agreement prior to meeting the patient. After reviewing this information, the clinicians joined a Zoom breakout room with the SP portraying their patient. Once the provider ended the first encounter, the portraying SP turned off their camera, and, to reflect the passage of time between visits, the moderator gave the clinicians the results of any testing they ordered and any information about the patient that had changed between the last and next visit. The provider indicated when they were ready to start the next encounter. This process was repeated between the second and third encounter.

### Data collection: semi-structured interviews

Immediately after they interacted with the SPs, each participant completed a one-on-one interview to reflect on and assess the experience, as well as to provide feedback on how the algorithms should ultimately be integrated into practices like theirs. Interviews were conducted by three experienced qualitative data specialists who work at Qualitative, Evaluation and Stakeholder Engagement Research Services (Qual EASE) at the University of Pittsburgh. Multiple interviewers conducted the interviews, because multiple interviews needed to be conducted at the same time following each SP session. Interviewers used a semi-structured interview guide developed by the research team that covered the following domains: (1) Assessment of their orientation to the algorithms, including training; (2) Assessment of their interaction with the SPs; (3) Assessment of and opinions on the algorithms; and (4) Description of how they thought the algorithms would operate in their practices, and how they could best be implemented there. Interviews were conducted on Zoom and recorded.

Questions and further probing were used to best assess how the algorithms could be implemented in their practices, which map onto several CFIR domains and constructs as shown in Table 2.

**Table 2:** Mapping of CFIR domains and constructs in interview guide

CFIR domain (definition in parenthesis)	Selected constructs from domain	Corresponding question(s)
Innovation (characteristics of “the thing” being implemented, i.e., the innovation)	Innovation design	What is your general impression of the algorithms? Probes: What, if anything, about them is useful? What, if anything, about them was unhelpful?
	Innovation adaptability	How would the description (or orientation) of the algorithms that we presented need to be tailored to your practice? What, if any, edits did you find yourself wanting to make to the algorithms?
Outer setting (the setting in which the inner setting exists, e.g., the health system and community in which the practice sits)	Local conditions	What clinic (or health system) level factors would make implementing these algorithms easy? What factors would make it difficult?
	Partnerships and settings	How, if at all, would insurance company policies impact your ability to implement these algorithms?
Inner setting (the setting in which the innovation will be implemented, e.g., the individual practice)	Work infrastructure	How would you fit the algorithms into your workflow at your practice? How would you like to use these algorithms in your day-to-day practice (if you would like to use them – if you wouldn’t like to use them, we’d love to hear about that too)?
	Relational connections	Next, I would like to talk about implementation of these algorithms in practices like yours. First, tell me about your practice. Probes: How many providers are there? How many patients are in it? How would you describe your patient base?
	Information technology infrastructure	What would be the benefits/drawbacks of incorporating the algorithms into the EHR? How would you do it?
Individuals (the roles and characteristics of individuals involved in or affected by implementation of the innovation)	Innovation recipients	How do you think patients will respond to these algorithms, and what types of things do clinicians need to do make this acceptable to patients? What would make it easy or difficult to implement these algorithms with a given patient?
	High-level leaders	How could we best get practice and PCP buy-in to implement these algorithms?
	Mid-level leaders	What would make it easy or difficult for individual PCPs to implement these algorithms?
Implementation process (the activities and strategies used to implement the innovation)	Tailoring strategies	This morning, we provided you with a brief orientation (or “introduction”), or description of the algorithms. What was good about the initial description? What could have been better? How would the description (or orientation) of the algorithms that we presented need to be tailored to your practice? How would you want doctors in your practice to be oriented [trained] to the algorithms? If these algorithms were implemented in your practice, what type of ongoing training, if any, would you and your colleagues want to receive? What kind of interactive assistance would be helpful? (Having a support person in the clinic? Over the phone?) What kind of patient-level support would be helpful? (i.e., What kind of support do you need for your patients in order for you to use the algorithms effectively?) (Infrastructure? Financial?)

Within one week of their completion, the qualitative methodologist associated with the project wrote a summary of each interview, which was forwarded to the study team so that they could begin to plan for implementation. Following that initial summary, interviews were transcribed verbatim with identifying details redacted. Under the supervision of the qualitative methodologist, experienced analysts at Qual EASE inductively developed a codebook reflecting the content of the interviews, with coding categories reflecting the four areas of the interview guide mentioned above. Use of the codebook was practiced on two transcripts by 2 Qual EASE coders, following which they both applied the codebook to the remaining 10 transcripts. Cohen’s Kappa statistics were used to assess intercoder reliability; the average kappa score was 0.8565, indicating “near perfect” agreement. The primary coder for the project then conducted a conventional content [31] and thematic analysis [32, 33], which was reviewed by the qualitative methodologist, and shared with the study team to better facilitate implementation planning.

### Data collection: development of implementation bundle

The final step to developing the implementation bundle—which included materials for initial training, an online algorithm interface, e-consultation support, and electronic health record (EHR) integration for the 6 algorithms—was to review notes from the structured interviews. The bundle was then drafted and reviewed by the PPABs and co-Is.

### Recruitment and study sample

Recruitment emails were sent to Community Medical Inc. (CMI). CMI is a network of 400 primary care and specialty physicians who practice throughout western and central Pennsylvania and provide care for over 495,000 patients. The practices cover a large geographic area; however, the network is predominantly in Allegheny County. Participants were required to be primary care clinicians at CMI practices and at least 18 years of age. Each of the clinicians were recruited to participate in two virtual patient evaluations followed by one-on-one interviews. The experience lasted approximately 4 h and clinicians were paid $1000 for their participation. We ultimately recruited 12 PCPs to participate in the virtual experience, which provided two perspectives for each of the 6 SP cases.

## Results

Table 3 summarizes the demographic characteristics of the clinicians participating in our study. All of our participants (100%) were trained as physicians with 33% specializing in Internal Medicine, while 66% specialized in Family Medicine during their residency. There was a prevalence of urban practitioners (58%), followed by those in suburban areas (42%), with an absence of participants from rural locales. We had 42% male and 58% female participants. The racial and ethnic composition of our study cohort is diverse, with White participants comprising the majority at 50%, followed by 33% of participants identifying as Asian. Additional categories encompass Hispanic, Latino, or Spanish origin of any race (17%), and two or more races (17%), with a nuanced representation of other racial and ethnic identities.

**Table 3:** Participant characteristics

*Training*	
Physician	12 (100%)
Nurse practitioner	0 (0%)
Physician assistant	0 (0%)
*Residency training*	
Internal medicine	4 (33%)
Family medicine	8 (66%)
Physical medicine and rehabilitation	0 (0%)
Other	0 (0%)
Did not complete a residency	0 (0%)
*Practice setting*	
Urban	7 (58%)
Suburban	5 (42%)
Rural	0 (0%)
*Gender*	
Male	5 (42%)
Female	7 (58%)
Other	0 (0%)
*Race/ethnicity*	
Black or African American	0 (0%)
White	6 (50%)
Asian	4 (33%)
Native Hawaiian or Other Pacific Islander	0 (0%)
American Indian/Alaskan Native	0 (0%)
Other	1 (8%)
Hispanic, Latino or Spanish Origin of any race	2 (17%)
Two or more races	2 (17%)

### Implementation support strategies

When asked about how algorithms should be implemented in practices like theirs, clinicians indicated that the orientation they had received to the algorithms would be a useful implementation support strategy. Other themes illustrating helpful implementation support strategies included (1) the importance of having the algorithm use endorsed by practice leadership, and of having a local “champion” who promoted their use; (2) integration of the algorithm workflow into practice EHRs; (3) practice and location-specific inputs into the algorithms, such that a suggestion to refer to a specialist come with a list of who, specifically, to refer to, or a suggestion to call security provide the practice-specific number for security; (4) access to specialists who could help interpret unclear or difficult-to read drug screens or suggest a particular course of action with a tricky patient.

Representative quotes supporting these themes, as well as the CFIR domains that they map to, are provided in Table 4. These findings were integrated into an implementation toolkit that included an initial training session followed by a suite of supports, including EHR integration, algorithm guidance hosted on a separate website with links to useful tools, and support for clinician participants via e-consultation.

**Table 4:** Quotes from semi-structured interviews

Theme	CFIR domain	Quotes
The importance of having the algorithm use endorsed by practice leadership, and of having a local “champion” who promoted their use.	*Innovation, outer setting, inner setting, individuals*	“If it comes from administration, from the top, you think it’s more validated. ... if it just comes from a pain practice, it’s less likely, because we don’t know if it just comes from their opinion ... if it’s been implemented at a specific practice already and has been useful and believed to be efficient, and is easily implementable, ... if somebody who does this and tells you, yes, this easily implemented, even despite all the things we have to do, this does help me make things faster, um, then it’s more likely for other people to look at it.” (Participant 11)
		“If you got the head of all the private practice providers to say this is our-what we’re gonna do, ... at least everyone would have the same set of tools, ... instead of sort of saying here’s a bunch of things, you know, do in your office how you will. ... while that gives flexibility, it also allows for a lot of variability. ... if there’s way too much variability, then, you know – like that last standardized patient said to me, I’ll just switch doctors. ... because they don’t like the answer that they’re given. So, if you knew that it was gonna be consistent, then maybe people would be a little less apt to do that.” (Participant 2)
Integration of the algorithm workflow into practice EHRs.	*Inner setting*	“If it becomes a smart set or an EPIC list or something like that where you have a check-off, if it’s a missed appointment issue and it goes into that segment of the algorithm versus the other pieces, of other substance use or taking opioids for symptoms other than pain and those kinds of things. I think having kind of a check-off box that leads you to the next [step] – and then it opens kind of the next step piece would be helpful.” (Participant 4)
		“I think converting these to templates in EPIC that we can instantly import to them into the chart. Because, no matter how good we are, these papers are going to get lost in a huge, massive pile of papers in the office. So, if I can ... just enter dot I G opiates, early appointment, early prescription, and gives me the questionnaire and-and I go over the questions, we’re good. ... Because our office hours are extremely, extremely tight. Every day there are new things that we need to add doing to our patients. While you are talking to them, talk about colon cancer screening. Um, you can easily spend half an hour convincing someone to take the COVID vaccine and… So, just-just so add-ons. So, if this is going to be a part of usually many other things in that visit, we need to be very time-efficient.” (Participant 5)
Practice and location-specific inputs into the algorithms, such that a suggestion to refer to a specialist come with a list of who, specifically, to refer to, or a suggestion to call security provide the practice-specific number for security.	*Inner setting, implementation process*	“It would need to have some specific numbers. So, things like call security. ... So, it’s not like I have to go look something up while I’m dealing with someone with an aggressive behavior, right? [and] when it says refer to specialist ... an addiction specialist number, chronic pain number, those listed out so, again, we’re not having to dig for them.” (Participant 3)
		“[We need direct referrals or embedded practitioners because] if you’re going through the algorithm and it says, you know, address underlying problems, and their underlying problem is uncontrolled anxiety, that’s what they need is therapy, and you don’t have it. ... And telling people to call the back of their card to figure out who takes their insurance is, like, archaic at this point.” (Participant 1)
Access to specialists who could help interpret unclear or difficult-to read drug screens, or suggest a particular course of action with a tricky patient.	*Outer setting, implementation process*	“It would be helpful to have better pain management resources, especially in terms of medication management. Somebody that I can reach out to and say, ‘hey, listen, can you eyeball the chart. ...what do you think?’ Like, [an] opioid management e-consult. They have that paradigm already for some other health conditions.” (Participant 12)
		“I think another potential place could be if the consultants that were calling for help saw a pattern of, like, they keep asking the same question that I keep answering, that would be a time to be like, maybe we need to re-educate this piece, because I keep getting the same call from different people. So, those-those folks might help steer directions of, like, where our-where there’s an education lapse.” (Participant 3)

### Simulation feedback from clinicians

We identified two themes related to the physicians’ encounters with the SPs: (1) clinicians found it useful to practice the algorithms with the SPs; (2) while clinicians applauded the skill of the SPs, they noted that not all actual patient counters go so smoothly. Each is presented in more detail below.

#### Clinicians found it useful to practice the algorithms with the SPs

Clinicians interviewed found it useful to practice the algorithms with the SPs. As will be discussed below, not all clinicians found the scenarios or SP reactions to be fully realistic. However, they did find practicing the algorithms in this way to be a useful way of learning the algorithms. As one provider put it:

It was a good chance to sort of get to look through the algorithm while I’m talking to them and sort of follow along. So, that was good to get familiar with the algorithm itself in a situation where you don’t feel like you’re with a real patient who you’re, like, ignoring to read through the algorithm.

Another provider similarly reflected:

So, that was really helpful, because this is sort of cut and dry of the way it’s written. And not until you’re in an actual patient scenario do you see some of the gray nuances. For example, one of the cases, the patient was having trouble sleeping secondary to pain. So, she was using her oxycodone in the evening to help with sleep, but it was related to pain. So, it wasn’t this clear-cut ‘I’m just using this to fall asleep at night.’ It was ‘I’m using this because at night my pain is worse which is affecting my sleep, so that’s why I’m using it.’ Which is a gray space. So, having the algorithm to sort of follow through and use as a guide let me make sure I’m asking all the right questions, let me make sure I’m offering all the other alternative things, was definitely beneficial.

#### While clinicians applauded the skill of the SPs, they noted that not all actual patient counters go so smoothly

Many clinicians described the practice session with SPs as being realistic or very similar to encounters with real patients. One provider described themselves as “shocked” at how realistic the SPs were, adding that “I felt very engaged in each of the scenarios. Like, they knew their background, they kind of were living the patient. I was really impressed... the scenarios were spot-on.” Other clinicians described the scenarios as “realistic situations that you can see in the office every day,” and “totally realistic.”

However, some clinicians described pointed differences with real life patient visits. For example, the following provider described that some of their actual patients would simply never agree to the treatment plans presented in the algorithms:

In the back of my mind I’m thinking of my actual patients who I’ve run into these instances and how this would go, and I don’t think it would’ve – it won’t go the way that it went with the SPs. Because it sometimes doesn’t matter how good your rapport is, they just aren’t gonna do what’s suggested... I think I run into much harder stops with some of my real non-SP patients.

Another clinician echoed this description, noting that:

My experience is that patients don’t normally accept what you say so easily. […] The interactions that I have with my patients are not anything like these, ‘cause these were very calm, very reasonable, willing to listen to you; they seemed to have a health literacy level that is well beyond a lot of the patients I deal with.

While these concerns were not voiced by every clinician, they were voiced by clinicians who experienced different scenarios with the SPs, indicating that patients may not always be agreeable to the actions suggested in the algorithm—and that that lack of agreement would be something that would need to be managed in an ongoing patient relationship, rather than disappearing at the end of the role play with the SP.

## Discussion

In this study, we used the SP methodology in combination with one-on-one interviews guided by CFIR to develop an implementation bundle for 6 algorithms designed to address common and challenging behaviors associated with LTOT. We found the use of the SP methodology to be a valuable tool for highlighting important components of an implementation bundle. Specifically, we found that an implementation bundle addressing (1) the importance of having the algorithm use endorsed by practice leadership, and of having a local “champion” who promoted their use; (2) integration of the algorithm workflow into practice EHRs; and (3) practice and location-specific inputs into the algorithms would be most effective in promoting the successful adoption and implementation of the EPBs for the LTOT algorithms. We also found that the SPs were realistic in their portray of patients with LTOT; however, it was noted that patients of the clinicians that participated in the simulations were likely to be more resistant to the adoption of the recommendations outlined by the algorithms than the SP portrayal. SPs are trained to recognize and reward participant skill, which may account for this observation.

Of methodological note in the realm of qualitative research: completing the interviews just after the SP interactions set an excellent stage for collecting qualitative data, likely because clinicians had just had a novel experience that was fresh in their minds. They could also talk about the details of the SP cases without concern for inappropriately describing actual patient cases in too much detail and contrast the SPs with their patients in general. This made for highly engaging interviews in which rapport building between interviewer and interviewee was more easily built. Additionally, interviews were conducted by qualitative research specialists who were not personally invested in the development of the algorithms or orientation to the algorithms, setting the stage for open and honest feedback.

## Discussion

In this study, we used the SP methodology in combination with one-on-one interviews guided by CFIR to develop an implementation bundle for 6 algorithms designed to address common and challenging behaviors associated with LTOT. Our findings underscore the value of the SP methodology in elucidating essential components of the implementation bundle. Specifically, we found that an implementation bundle addressing (1) the importance of having the algorithm use endorsed by practice leadership, and of having a local “champion” who promoted their use; (2) integration of the algorithm workflow into practice EHRs; and (3) practice and location-specific inputs into the algorithms would be most effective in promoting the successful adoption and implementation of the EPBs for the LTOT algorithms. We also found that the SPs were realistic in their portrayal of patients with LTOT; however, it was noted that patients of the clinicians that participated in the simulations were likely to be more resistant to the adoption of the recommendations outlined by the algorithms than the SP portrayal.

Of methodological note in the realm of qualitative research: completing the interviews just after the SP interactions set an excellent stage for collecting qualitative data, likely because the experience was fresh in their minds. They could also talk about the details of the SP cases without concern for inappropriately describing actual patient cases in too much detail and contrast the SPs with their patients in general. This made for highly engaging interviews in which rapport building between interviewer and interviewee was more easily built. Additionally, interviews were conducted by qualitative research specialists who were not personally invested in the development of the algorithms or orientation to the algorithms, setting the stage for open and honest feedback.

Despite the merits of the SP approach in examining EBP implementation, several limitations warrant consideration. The applicability of SP methodology to diverse practices and various points in care management raises questions about its universal relevance. The effectiveness or practicality of SPs for EBP training and adoption may vary across different clinical settings, requiring careful consideration when extrapolating findings to practices with distinct characteristics or specific care management points.

The selection of long-term opioid therapy (LTOT)-related care as a case study introduces a contextual limitation. While SP methodology effectively addresses concerns within LTOT-related care, the transferability of findings to other healthcare scenarios might be constrained. The unique nature of LTOT-related care may not fully capture challenges present in different medical specialties or care contexts.

Additionally, while the goal of this manuscript is to illuminate the SP methodology, our study's findings may not be universally generalizable, considering factors such as regional variations in healthcare practices and differing levels of familiarity with EBP implementation. The dynamic nature of clinical practice introduces a limitation in capturing all potential scenarios through SP methodology. Clinician encounters with patients can vary widely, and SPs may not fully replicate the complexity of real-world situations.

Overall, this study demonstrates the potential of using the SP methodology guided by the CFIR framework to develop effective implementation strategies for improving care in real-world healthcare settings. The use of SPs allowed the research team to observe the EBP in practice with feedback from end-users with experience from different health care clinics. The CFIR framework provided a comprehensive approach to guiding the development of an implementation bundle that addressed the multiple factors that influence EBP implementation. The study’s success prompts further exploration of whether the developed implementation bundle correlates with increased EBP adoption levels to further validate the use of SPs for this purpose.

## Data Availability

The dataset supporting the conclusions of this article is available from the corresponding author on reasonable request.

## Appendix: Instructions for participants

Dear Participant,

Thank you for participating in our study of opioid misuse in primary care.

In this exercise, you will encounter 2 different simulated patients played by standardized patients (SPs) of the SP Program of the University of Pittsburgh School of Medicine. The purpose of these visits is to help us study clinical algorithms for managing opioids.

Therefore, please be aware of the following expectations:

- For this simulated scenario, each of these patients were started on opioid treatment by one of your partners who recently left your practice (Dr. Kia Williams). You may not have started opioids if it were up to you, but they have already been started and have an opioid agreement with this practice. Therefore, please focus your time on the algorithms and not on whether the patient should/should not have been started on opioids.
- You will see each patient in 3 separate “telemedicine” visits via Zoom. Therefore, you do NOT need to perform a physical examination for these visits.
- The “visits” will occur in break out rooms on the Zoom platform. The 1^st^ visit will be to establish care with you after Dr. Williams has left the practice. The next 2 visits will be follow-up visits.
- For each scenario, there will be a “moderator” in the breakout room with you and the SP. The moderator’s camera will be off. This person will be helping with timing of the visits, and they will post updates about the patient’s case before each visit in the chat section.
- Therefore, please enable the chat on your screen.
- Also, please “hide nonvideo participants”, so the presence of the moderator is not a distraction for you as you conduct the visits. (If you need help in how to do this, please ask, so a team member can walk you through the steps)
- In the interest of transparency, the moderators are also SPs. They are not clinicians.
- After you are done with the visits, you will meet with researchers from the study to debrief your experience.

Timing of the whole activity:

- Orientation: 30 min
- Encounter with 1^st^ patient: 60 min
- Break (including time to prepare for 2^nd^ patient): 15 min
- Encounter with 2^nd^ patient: 60 min
- Debrief with researchers: 75 min

Timing of your patient visits: You have 1 h for each session, which includes 3 distinct visits with the same patient. You will see timing banners at 15-min increments, and a 5-min warning. How you divide the time between the three visits is up to you.

If it would help you communicate with the patients in the simulation, here is some information about Dr. Kia Williams:

- Dr. Williams recently left your practice to be closer to her family in South Carolina. Her father’s dementia has been worsening, and she wanted to be closer to her family in this time. As your partner, she was well liked by your colleagues, the staff, and her patients. She was an excellent doctor and a friend.
- Prior to meeting each patient, we will share Dr. Williams’ last progress note with you. You will have time to review that information before starting the first visit. This note will have information about what work up has been done and what pain treatments have been tried.
- The patients are aware of what has been tried, and they can answer these questions, but for the sake of this study, you do not need to explore this in great detail given the limited timeframe of each visit.
- Dr. Williams had an opioid agreement with each of the patients you will meet today. This will also be shared with you in case you need to reference it during the sessions.

Thank you

## Abbreviations

CFIR: Consolidated framework for implementation research
CMI: Community Medical Inc.
Co-I: Co-investigator
EBP: Evidence-based practice
EHR: Electronic health record
LTOT: Long-term opioid therapy
PCP: Primary care physician
PPAB: Patient-provider advisory board
SP: Simulated patient
